# Group‐based trajectory modeling of body mass index and body size over the life course: A scoping review

**DOI:** 10.1002/osp4.456

**Published:** 2020-09-29

**Authors:** Vanessa De Rubeis, Alessandra T. Andreacchi, Isobel Sharpe, Lauren E. Griffith, Charles D. G. Keown‐Stoneman, Laura N. Anderson

**Affiliations:** ^1^ Department of Health Research Methods, Evidence, and Impact McMaster University Hamilton Ontario Canada; ^2^ Applied Health Research Centre Li Ka Shing Knowledge Institute St. Michael's Hospital University of Toronto Toronto Ontario Canada; ^3^ Division of Biostatistics Dalla Lana School of Public Health University of Toronto Toronto Ontario Canada; ^4^ Child Health Evaluative Sciences The Hospital for Sick Children Research Institute Toronto Ontario Canada

**Keywords:** body weight, growth mixture modeling, latent class growth analysis, life course

## Abstract

**Background:**

Group‐based trajectory modeling has been applied to identify distinct trajectories of growth across the life course. Objectives of this study were to describe the methodological approaches for group‐based modeling of growth across the life course and to summarize outcomes across studies.

**Methods:**

A scoping review with a systematic search of Medline, EMBASE, CINAL, and Web of Science was conducted. Studies that used a group‐based procedure to identify trajectories on any statistical software were included. Data were extracted on trajectory methodology, measures of growth, and association with outcomes.

**Results:**

A total of 59 studies were included, and most were published from 2013 to 2020. Body mass index was the most common measure of growth (*n* = 43). The median number of identified trajectories was 4 (range: 2–9). *PROC TRAJ* in SAS was used by 33 studies, other procedures used include *TRAJ* in STATA, *lcmm* in R, and Mplus. Most studies evaluated associations between growth trajectories and chronic disease outcomes (*n* = 22).

**Conclusions:**

Group‐based trajectory modeling of growth in adults is emerging in epidemiologic research, with four distinct trajectories observed somewhat consistently from all studies. Understanding life course growth trajectories may provide further insight for population health interventions.

## 1 BACKGROUND

The prevalence of obesity has nearly tripled globally over the past 40 years.^1^ Specifically, in children, 18.5% are considered to have obesity, which is concerning as this may impact health‐related outcomes, including obesity, later in life.^2^ It is important to understand the life course patterns or trajectories of obesity as these may differentially impact health related outcomes.^3^ It is common for researchers to measure obesity at a single time‐point; however, a single time‐point may not adequately represent disease risk and etiology and does not address the potential heterogeneity of growth patterns across the life course.^4^ Within populations, heterogeneous patterns of body mass index (BMI) or body size over the life course exist. Identifying homogeneous groups that have followed similar trajectories can provide important insight for both treatment and prevention and for understanding disease risk.^5^ Specific groups of people that share similarities in growth patterns may be differentially at risk for development of chronic diseases later in life.

Several approaches can be used to measure growth over the life course. Characterization of growth can be done using growth curves that assess individual change over time, or through group‐based methods, which identify groups of individuals who share underlying characteristics.^6^, ^7^ The use of group‐based procedures to categorize patterns of growth is an emerging method in epidemiology; however, the methods associated with generating group‐based trajectories vary. Two common methods include latent class growth analysis or growth mixture modeling which are finite mixture modeling approaches that identify groups of individuals who share underlying characteristics.^8^ The advantages of using group‐based procedures to understand growth over the life course are the potential identification of sensitive or critical periods of exposure. There are periods of accelerated growth in childhood and the incidence and remission of obesity changes with age.^9^ Understanding the impact of accumulation of risk or change in risk of obesity across the life course may help to better understand the risk of disease various chronic diseases.^6^, ^7^

A previous systematic review of group‐based trajectory modeling for BMI trajectories only in childhood, starting at birth, found that most studies identified three or four distinct trajectories; however, there were several inconsistencies in terms of methodologies used to identify trajectories.^10^ A limitation of the previous review is that it only included studies that had a measure of growth at birth and only those that used BMI as the anthropometric measure. There have been no reviews that included measures of growth for adults over the age of 18. Thus, the primary objective of this study is to review the methodological approaches and results of group‐based modeling studies of growth across the life course. A secondary objective is to describe the outcomes associated with growth trajectories.

## 2 METHODS

### 2.1 Study design

A systematic scoping review was conducted. This protocol review was registered with PROSPERO (CRD‐42019129356). The preferred reporting items for systematic reviews and meta‐analyses extension for scoping reviews guidelines were followed for the reporting of this study.

### 2.2 Eligibility criteria

Studies were included if a group‐based approach to construct trajectories of anthropometric measures were used. Inclusion criteria were that all studies had at least three repeated anthropometric measures taken over a period of at least 1 year, and at least 1 measure had to be recorded while the participant was >18 years of age. According to Medical Subject Headings^11^ and World Health Organization,^12^ growth is defined as a gradual increase or development in cells that results in changes in body weight or height. Therefore, any anthropometric measures to assess growth (e.g., BMI, height, weight, waist circumference, body size, waist‐to‐hip, and skinfold thickness) were included. Any exposures or outcomes evaluated in relation to the growth trajectories were eligible for inclusion into the review. Studies were excluded if they focused on a specific clinical population, for example, only people with diabetes, spinal cord injuries, or who were pregnant. Studies that looked at growth velocity or modeled weight gain or weight loss after a medical procedure were also excluded. Any year of publication or study design were included; however, only studies published in English and primary studies were included (abstracts and review papers were excluded).

### 2.3 Search strategy

A systematic search was conducted in August 2020 using four databases: Medline, EMBASE, CINAL, and Web of Science. The search strategy was developed with assistance from health research librarians at McMaster University. Search strategies were developed and modified for the specific criteria of each database. Search terms fell into two categories: latent class growth modeling and anthropometric measures (BMI, weight, etc.). The sample search for EMBASE can be found in Table 1. Search strategies for the remaining databases can be found in the Tables A1–A3. The reference lists of included studies were reviewed to determine any further studies that were eligible for inclusion into the study.

**TABLE 1:** EMBASE search strategy

1	“Latent growth model*”.mp OR “latent class growth mixture model*”.mp OR “growth mixture model*”.mp OR “latent growth model*”.mp OR “latent class growth analysis”.mp OR “latent class growth analyses”.mp OR “group based trajectory model*”.mp OR “group based trajectory analysis”.mp OR “group based trajectory analyses”.mp OR “group based model*”.mp OR “latent growth mixture model*”.mp OR “group based trajectory*”.mp
2	Growth/OR obesity/OR anthropometry/OR exp body mass/OR exp anthropometric parameters/OR exp body weight/
3	1 AND 2
4	Limit 3 to (human and English language)
	547

### 2.4 Study selection

Once searches were conducted in all databases, studies were imported into Covidence. Covidence is a web‐based software used to maintain records throughout the various stages of conducting a systematic review.^13^ All duplicates were identified and then removed prior to beginning screening. Studies were screened at title and abstract level, and then at full text by three independent reviewers (Vanessa De Rubeis, Alessandra Andreacchi, Isobel Sharpe). Conflicts at both title and abstract level and full‐text level were resolved by the reviewers and a final decision was then made regarding inclusion of the study.

### 2.5 Data extraction

All eligible studies had data extracted by two independent abstractors (Vanessa De Rubeis, Alessandra Andreacchi, Isobel Sharpe). Any conflicts that arose during data extraction were resolved by a third reviewer (Vanessa De Rubeis, Alessandra Andreacchi, Isobel Sharpe). A data extraction template on Microsoft Excel was used to organize the information extracted from each study. Data on the general characteristics of the study, including the author, year of publication, name of study, sample size, and population were extracted. Data were also extracted on the methodology used to generate trajectories, including statistical modeling methods, statistical software used, and model fit criteria. Trajectory details were also extracted, which included the number of trajectories, the shape of trajectories (e.g., cubic, quadratic), names of trajectories, and proportion of people in each trajectory. The measures of growth used to identify the trajectories, the number of measures, and the period of life course which the trajectories encompass were also extracted. Finally, details regarding if the growth trajectories were considered as an exposure or outcome were extracted, and in studies where growth trajectories were the exposure, associations with outcomes were extracted.

## 3 RESULTS

**FIGURE 1:**
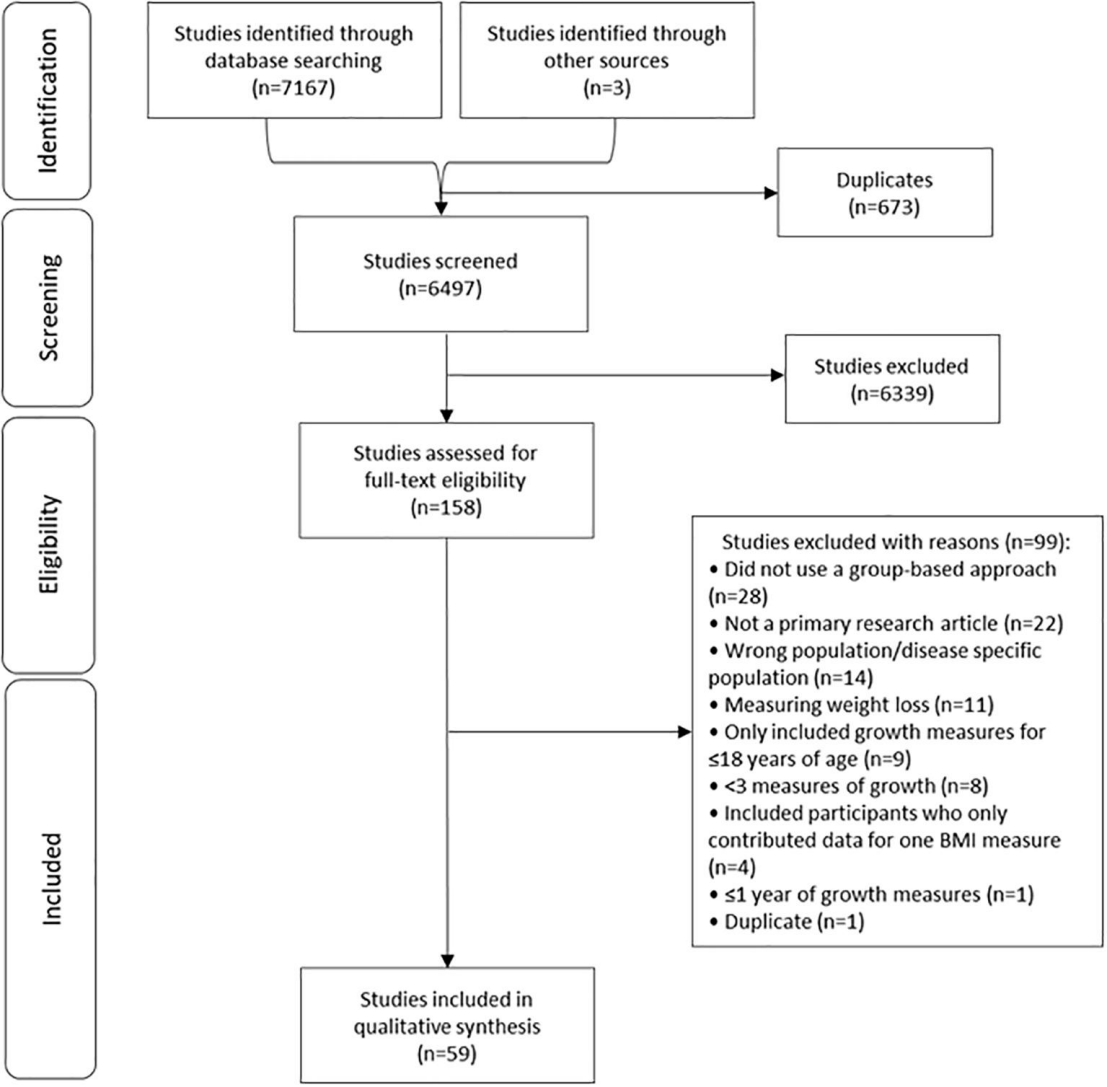
Preferred reporting items for systematic reviews and meta‐analyses flow chart

A total of 7170 studies were identified from the search and three additional studies were identified from the reference lists of the included studies. Six hundred and seventy‐three duplicates were identified and removed. There were 6497 studies that were screened at title and abstract level and 158 of these studies were screened for eligibility at full‐text level. A total of 59 papers met the inclusion criteria and were included in this review. A detailed description of the screening process can be found in Figure 1.

### 3.1 Description of studies

A summary of study characteristics can be found in Table 2. Of the 59 papers included in this review, 40 unique study populations were represented. For instance, there were six studies (10%) from the Nurses' Health Study and Health Professionals Follow‐Up Study^14^, ^15^, ^16^, ^17^, ^18^ and four studies (7%) from the US National Longitudinal Survey of Youth (NLSY).^14^, ^15^, ^16^, ^17^, ^18^ Most of the papers included North American populations, with 28 (47%) studies from the United States, five (8%) studies from Canada, and one study from Mexico. The remaining studies were conducted in Asia (*n* = 8, 14%), Australia and New Zealand (*n* = 3, 5%), and 14 studies (24%) were published from European countries. Most studies were published from 2013 to 2019 (*n* = 55). Few studies were restricted to males (*n* = 4)^14^, ^15^, ^16^, ^17^, ^18^ or females (*n* = 8)^14^, ^28^, ^29^, ^30^, ^31^, ^32^, ^33^, ^34^ only. Of the 47 studies that included both males and females, 18 studies reported trajectories only stratified by sex. The most common study design used was a cohort study (*n* = 55), three studies were case‐control designs^27^, ^30^, ^35^ with measures of recalled body size from different age periods and one study was a randomized control trial.^27^, ^30^, ^35^ Table A4 in the Appendix outlines more detailed characteristics of the included studies.

**TABLE 2:** Characteristics of included studies (*n* = 59)

Characteristics	*N* (%)
Place of publication	
Australia/New Zealand	3 (5)
Asia	8 (14)
Europe	14 (24)
North America	34 (58)
Year of publication	
2017–2020	33 (56)
2013–2016	22 (37)
2009–2012	4 (7)
Study design	
Cohort study	55 (93)
Case‐control study	3 (5)
Randomized trial	1 (2)
Sex	
Male only	4 (7)
Female only	9 (15)
Both	46 (78)
Sample size	
≥10,000	16 (27)
5000–<10,000	18 (31)
≤5000	25 (42)
Software used to generate trajectories	
Mplus	11 (19)
R	4 (7)
SAS	33 (56)
Stata	11 (19)
Number of trajectories^a^	
2	9 (15)
3	13 (22)
4	33 (56)
5	19 (32)
>6	8 (14)
Anthropometric measure used^a^	
Body shape^b^	11 (19)
Body weight	2 (3)
Self‐reported BMI	26 (44)
Measured BMI	16 (27)
Other^c^	6 (10)
Period of life	
Birth to adulthood	1 (2)
Childhood to adulthood	21 (36)
Adulthood	37 (63)
Stratified trajectories	
Sex stratified	19 (32)
N/A	40 (68)
Mean number of trajectories (SD)	4.2 (1.4)
Mean number of growth measures used^d^ (SD)	6.2 (2.7)

Abbreviation: BMI, body mass index; N/A, Not applicable; SD, Standard deviation.

^a^ Does not equal to 100% since studies included trajectories for multiple measures or stratified trajectories.

^b^ Body shape assessed used somatotypes or body silhouettes.

^c^ Body fat percentage, lean mass, total body fat mass, skinfold thickness, waist circumference.

^d^ Six studies did not provide exact number of measures used and only reported a range, therefore were not included in mean calculation.

### 3.2 Growth measures used and period of life

Table 3 summarizes the growth measures and methods that were used to identify trajectories. BMI was the most commonly used growth measure (*n* = 43). Two studies estimated distinct trajectories for more than one measure of growth.^26^, ^36^ Of the 43 studies that used BMI, 27 (63%) relied on self‐report, and the remaining 16 (37%) used directly measured height and weight by trained research assistants. Eleven studies used body shape as the growth measure. To measure body shape, studies used pictures or somatotypes, ranging from lean to overweight, which assist in recall of past or current body size. Two studies^26^, ^36^ used measured weight (unadjusted for height) to estimate trajectories. Body fat percentage,^26^, ^36^ total lean mass,^26^, ^36^ total body fat mass,^26^, ^36^ skinfold thickness,^26^, ^36^ and waist circumference^26^, ^36^ were also used to estimate trajectories in one study each. One study^40^ modeled percent change of BMI from the baseline measure at 20 years of age. No studies used height only as a measure growth.

**TABLE 3:** Detailed description of trajectory methodology of included studies (*N* = 59)

Author, year	Trajectory method	Model fit criteria	Shape	Software	Number of trajectories	Trajectory name (%)	Resolving trajectory	Anthropometric measure	Period of life	Number of measurements	Duration of measurements
Adane, 2018^28^	N/A	BIC A priori knowledge about BMI trajectories in adults Size of trajectory group (5% cutoff) Average group membership probability Meaningfulness of groups for analyses	N/A	SAS—proc traj	3	Normative (61.2) Chronically overweight (30.7) Chronically obese (8.1)	No	Self‐reported BMI	Adulthood—preconception (25–32 years)	3	6–7 years
Ahanchi, 2019^51^	LCGMM	BIC AIC Adjusted LMR likelihood ratio test Class size Interpretability Entropy Posterior probability	Quadratic	Mplus	Outcome: Incident high plasma glucose: Females: 2 Males: 2 Incident high bp: Females: 3 Males: 2	Incident high plasma glucose Females: Overweight to late obese (12.6) Normal weight (87.4) Males: Overweight to late obese (12.6) Normal weight (87.4) Incident high bp Females: Overweight (4.1) Normal weight (91.7) Overweight to late obese (4.2) Males: Normal weight (74.0) Overweight (26.0)	No	Measured BMI	Adolescence (12 years)–young adulthood (20 years)	5	∼16 years
Amadou, 2014^30^	Discrete mixture model	BIC Number of people in each group	Quadratic and cubic function	SAS—proc traj	5	Group 1: Constantly low (14.4) Group 2: Constantly mid‐range (40.3) Group 3: Moderate increase (20.3) Group 4: Strong increase (21.9) Group 5: Constantly high (3.2)	No	Body silhouettes	Childhood (6–11 years of age)–midadulthood (25–35 years of age)	6	∼27 years 24–63 years of age
Botoseneanu, 2013^52^	SPMM	Posterior probabilities Maximum likelihood estimation Censored normal distribution BIC	Quadratic	SAS—proc traj	5	Trajectory 1: Normal increasing (19.9) Trajectory 2: Overweight, increasing (43.8) Trajectory 3: Borderline obese, increasing (25.4) Trajectory 4: Obese, increasing (8.9) Trajectory 5: Morbidity obese, with decelerating gain (2.1)	No	Self‐reported BMI	Adulthood (∼55 years)–older adulthood	4–11	Mean 5.5 years (2.2–11.3 years)
Buscot, 2018^49^	LCGMM	BIC Class membership Poster probabilities (>0.7) Classification to assess goodness‐of‐fit of competing models	Quadratic	R—lcmm	6	Trajectory 1: Stable normal (55.2) Trajectory 2: Resolving (1.6) Trajectory 3: Progressively overweight (33.4) Trajectory 4: Progressively obese (4.2) Trajectory 5: Rapid overweight/obese (4.3) Trajectory 6: Persistent increasing overweight/obese (1.2)	Yes (class 2 crosses over)	Measured BMI	Childhood–adulthood (6–49 years of age)	16	31 years
Clarke, 2010^69^	Generalized growth mixture modeling	BIC AIC Statistically significant LMR likelihood ratio test Posterior probabilities Differences in mean outcomes across classes	Linear	Mplus—FIML	2	Class 1: Normative weight gain (80.9) Class 2: Persistently overweight (19.1)	No	Self‐reported BMI	Early adulthood (19–35 years)	7	55 years
Clarke, 2013^58^	Generalized growth mixture modeling	BIC AIC Statistically significant LMR likelihood ratio test Posterior probabilities	Linear and quadratic	Mplus—FIML	2	Class 1: Normative weight gain (78) Class 2: Persistently overweight (22)	No	Self‐reported BMI	Early adulthood (19/20 years–29/30 years)	3 or more measures including baseline and year 10	10 years
Dai, 2019^72^	GBTM	BIC Posterior probability (>0.7) >5% of total sample	Linear	STATA—traj plugin	4	Low (19.6) Moderate (33.4) Moderate‐high (33.4) High (13.6)	No	Measured BMI	Midadulthood (32–57 years)	6	5 years
De rubeis, 2019^35^	LCGMM	BIC Posterior probabilities A priori knowledge Significance of polynomial terms	Quadratic	SAS—proc traj	5	Class 1: Stable normal weight (38.9) Class 2: Progressively overweight (42.2) Class 3: Persistent overweight (12.6) Class 4: Progressive obesity (4.2) Class 5: Persistent obesity (2.1)	No	Self‐reported recall BMI	Early adulthood (20s)–older adulthood (50s–60s)	4	∼40 years (teenage years to 80s)
Elrashidi, 2016^60^	Nonparametric hill climbing algorithm	Calinski and Harabatz criterion	N/A	SAS—proc traj	4	Normal (26.9) Overweight (36.2) Obese (26.4) Severely obese (10.5)	No	Measured BMI	Early adulthood—adulthood (>18 at baseline– < 51 at final measurement)	12	4 years
Elsenburg, 2017^41^	Growth mixture modeling	Log likelihood AIC BIC Adjusted BIC Shape of trajectories Percentage of participants Models with entropy near >1% were considered good	Linear and quadratic	Mplus—FIML	3	Normal weight (75.1) Late onset overweight (20.1) Early onset overweight (4.8)	No	Measured BMI	Childhood (10–12 years)–early adulthood (21–23 years)	5	13 years
Fagherazzi, 2013^31^	Nagin's approach to group based modeling	BIC Percentage of people in each category	Cubic	SAS—proc traj	6	T1. Low increase (40) T2. Moderate increase (22.7) T3. Strong increase at menarche then decrease (9.3) T4. Strong increase (5.5) T5. Constantly mid‐range (17.0) T6. Constantly high (5.5)	Yes (T3 resolves after menarche)	Body shape	Childhood (8 years)–midadulthood (35–40 years)	4	32 years
Fagherazzi, 2015^32^	Nagin's approach to group based modeling	BIC Percentage of people in each category	Cubic	SAS—proc traj	6	T1. Constantly small body size (19) T2. Modest increase in body size at puberty (30) T3. Midrange body size (20) T4. Sharp increase in body size at puberty (3) T5. Upper midrange body size (23) T6. Constantly large body size (5)	No	Body shape	Childhood (8 years)–midadulthood (35–40 years)	4	32 years
Fan, 2019^66^	LCGMM	BIC decreased by at least 20 High mean posterior class membership probability (>0.65) High mean posterior probability (>0.70)	Cubic	R—lcmm	4	Low‐stable (45.8) Medium‐increasing (43.4) High‐increasing (8.9) Sharp‐increasing (1.9)	No	Measured BMI	Young adulthood (20–40 years)	3 or more	7 years
Hang, 2018^14^	Group‐based trajectory modeling	Optimal number of groups Shape of trajectories Censored normal model with polynomial function of age Posterior probability (>0.70)	Cubic	SAS—proc traj	5	Lean stable (22.9) Medium stable (26.5) Medium marked increase (35.5) Lean moderate increase (23.2) Lean marked increase (29.2)	No	Body shape	Children to older adulthood (5–60 years of age)	9	16 years
Ho, 2019^70^	Group‐based analysis	BIC	N/A	SAS—proc traj	4	Low‐normal weight group (20.3) High‐normal weight group (44.7) Overweight group (28.4) Obesity (6.6)	No	Self‐reported BMI	Older adulthood (average 61 years)	3	8 years
Huang, 2013^80^	Group based dual trajectory modeling	BIC AIC Posterior probability	Linear	SAS—proc traj	3	Low (72.1) Increased (14.6) High (13.3)	No	Self‐reported BMI	Early adulthood (20–24 years)	9	55
Islam, 2019^46^	LCGM	BIC Posterior probability (>80%) Significance of polynomial terms Group membership (>5%)	Linear and quadratic	SAS—proc traj	4	Normal stable (22.4) Low normal‐normal stable (44.1) Overweight‐obese (27.2) Low normal‐normal overweight (6.3)	No	Measured BMI	Childhood (6 years of age)–older adulthood (80 years of age)	6	11 years (19–30)
Ito, 2020^34^	Group‐based trajectory modeling	N/A	N/A	SAS—proc traj	4	Remained normal (82.3) Remained overweight (10.5) Gained weight (5.4) Lost weight (1.7)	Yes	Self‐reported BMI	Midadulthood (40s)—Older adulthood (>60 years)	6	10 years
Jayne, 2019^53^	GBTM	BIC Number of trajectories Order of polynomial terms Add complexity of trajectories Stability of estimates Percentage in each trajectory	N/A	Stata—traj procedure	4	Males: Increasing (11.1) Inconstant (21.1) Constant (60.6) Decreasing (7.2) Females: Increasing (10.6) Inconstant (22.4) Constant (60.6) Decreasing (7.0)	Yes	Measured BMI	Adulthood (3‐year duration)	5	3 years
Jeon, 2019^39^	GBTM	BIC Posterior probability (>0.7)	Linear quadratic Cubic	SAS—proc traj	5	Group A: Very low‐stable (12.8) Group B: Low‐stable (28.4) Group C: Moderate‐stable (33.2) Group D: Elevated‐increasing (20.4) Group E: High‐increasing (5.2)	No	Waist circumference	Midadulthood (>40 years)	4	6.2 years
Jun, 2012^15^	General growth mixture modeling	BIC Adequate sample size Posterior probability	N/A	Mplus	4	Group 1: Slow weight gain trajectory (61.8) Group 2: Moderate‐weight‐gain trajectory (22.4) Group 3: Rapid‐weight‐gain trajectory (14.0) Group 4: obese‐to‐overweight trajectory (1.8)	Yes (obese to overweight trajectory)	Self‐reported BMI	Early adulthood (25–29 years) –older adulthood (55–59 years)	7	74 years
Kakoly, 2017^33^	GBTM	N/A	N/A	STATA	3	1. Low‐stable group (63.8) 2. Moderately rising group (28.8) 3. High‐rising group (7.4)	No	Self‐reported BMI	Early adulthood (20–36 years)	5	16 years
Kelly, 2017^25^	LCGMM	BIC Number of participants in each group (>1%)	Linear and quadratic	SAS—proc traj	5	Stable normal (33) Normal to overweight (47) Stable overweight (10) Normal to obese (7) Overweight to obese (3)	No	Self‐reported BMI	Early adulthood (20 years) –older adulthood (62.5 years)	3	40 years (20 years to current, mean 62 years)
Kuchibhatla, 2013^64^	Generalized mixture modeling	AIC BIC Sample size adjusted BIC Entropy Condition number LMR LRT	Linear and quadratic	Mplus—GMM	3	Class 1: Mildly overweight class (65.1) Class 2: Obese class (7.3) Class 3: High normal class (27.6)	No	Self‐reported BMI	Older adulthood (baseline to 10 years follow‐up; 65–75 years)	4	10 years
Kvoerner, 2018^18^	GBTM	BIC Posterior probability (>0.7) Odds of correct classification (OCC) (>0.5)	Quadratic	SAS—proc traj	5	Males: Lean‐stable (29) Lean‐moderate increase (19) Lean‐marked increase (25) Medium‐stable/increase (20) Medium‐marked increase (7) Females: Lean‐stable (34) Lean‐moderate increase (23) Lean‐marked increase (19) Medium‐stable/increase (15) Medium‐marked increase (10)	No	Somatotype (for some measures, converted BMI to somatotype)	Childhood (5 years)–older adulthood (60 years)	9	18 years
Kwon, 2015^38^	Group‐based trajectory analyses	Average posterior probability (>0.7) OCC (>0.5) Proportion of sample assigned to group, similar to proportion assigned by model, 99% Confidence Intervals (CIs)	Quadratic	STATA—TRAJ	4	Males: 31.1 43.6 14.6 10.7 Females: 23.8 234.8 28.6 12.8	Yes (only in males)	Body fat percentage	Childhood (5 years)–young adulthood (19 years)	7	11 (12–23 years)
Laddu, 2017^26^	GBTM	BIC A priori knowledge	Quadratic	STATA—TRAJ	Body mass: 8 Total body fat: 5 Lean mass: 6	Body mass: Group 1: 5 Group 2: 19 Group 3: 26 Group 4: 22 Group 5: 16 Group 6: 8 Group 7: 3 Group 8: 1 Total body fat: Group 1: 36 Group 2: 28 Group 3: 15 Group 4: 15 Group 5: 5 Lean mass: Group 1: 8 Group 2: 23 Group 3: 30 Group 4: 23 Group 5: 13 Group 6: 4	Yes	Body weight (kg) Total body fat mass (kg) Total lean mass (kg)	Older adulthood (6.9 years (does not indicate ages but ≥65 at baseline mean = 73.7 [5.9])	3	6.9 years total follow‐up (visit 2 and 3 were an average 4.6 and 6.9 years after visit 1/baseline)
Lavalette, 2020^27^	Group‐based trajectory modeling	BIC Posterior probabilities (>0.7) 1% of participants within each traj	Linear Quadratic Cubic	SAS—proc traj	5	Stable normal (36.3) Normal BMI to overweight (28.6) Growing overweight (23.6) Normal BMI to obesity (7.6) Overweight to obesity (3.9)	No	Self‐reported BMI	Young adulthood (20s)–older adulthood (>70 years)	5 (2–6)	∼50 years
Lisan, 2018^47^	GBTM	BIC Posterior probabilities	N/A	SAS—proc traj	5	Lean stable (31.9) Lean increase (11.1) Lean‐marked increase (16.1) Moderate stable (32.5) Heavy stable (8.4)	No	Body silhouettes	Childhood (8 years) –midadulthood (45 years)	5	37 years
Lisan, 2019^48^	GBTM	BIC Posterior probabilities	N/A	SAS—proc traj	9	Heavy‐stable (8.1) Moderate‐stable (32.5) Lean‐stable (32.7) Lean‐increase (11) Lean‐marked increase (15.7)	No	Body silhouettes	Childhood (8 years)–midadulthood (45 years)	5	37
Malhotra, 2013^19^	GBTM	BIC Significance of polynomial terms Group membership probability Posterior probability (entropy) Number of people in each group	Linear	SAS—proc traj	5	Males: Normal weight in 1990 (46.2) Overweight in 1990 (41.1) Obese class I in 1990 (9.8) Obese class II in 1990 (1.8) Obese class III in 1990 (0.8) Females: Normal weight in 1990 (63.2) Overweight in 1990 (22.9) Obese class I in 1990 (9.0) Obese class II in 1990 (2.8) Obese class III in 1990 (2.2)	No	Self‐reported BMI	Young adulthood (25 years)–midadulthood (33 years)	Average 8.76, maximum 11	18 years
Nonnemaker, 2009^20^	GGMM	Adjusted BIC LMR test Average probability of class membership, entropy Shape of growth trajectories to assess validity ‐ is it consistent with a priori theory	Quadratic	Mplus	4	Class 1: High risk of becoming obese by young adulthood (4.3) Class 2: Moderate to high risk for becoming obese (15.9) Class 3: Low to moderate risk for becoming obese by young adulthood (35.6) Class 4: Low risk for becoming obese by young adulthood (44.1)	No	Self‐reported BMI	Childhood (12 years)–early adulthood (23 years)	7	16 years
Ostbye, 2011^21^	LCGM	A priori knowledge Model fit statistics BIC Significance of polynomial terms Group membership probability Posterior probability (entropy)	Linear and quadratic	SAS—proc traj	4	Normal weight (35) Overweight (41.2) Late adulthood obesity (19) Early adulthood obesity (4.2)	No	Self‐reported BMI	Young adulthood (18 years)–midadulthood (49 years)	10	30 years
Oura, 2019^50^	LCGM	BIC Posterior probabilities (>0.7) Sufficient group sizes Clinical significance of the models	Linear and quadratic	SAS—proc traj	3	Males: Group 1: Stable slim (30.7) Group 2: Stable average (59.9) Group 3: Early onset overweight (9.4) Females: Group 1: Stable slim (57.2) Group 2: Stable average (37.4) Group 3: Early onset overweight (8.1)	No	Measured BMI (*z*‐score)	Birth (0 years)–midadulthood (46 years)	5	46
Petrick, 2017^59^	Latent class group‐based mixture model analysis	BIC Posterior probability (>1%)	Quadratic	SAS—proc traj	4	Stable normal BMI (42.8) Normal BMI to overweight (43.4) Normal BMI to obese (11.7) Overweight to obese (2.0)	No	Self‐reported BMI	Early adulthood (20 years)–older adulthood (mean	3	59 years
Reinders, 2015^63^	Group‐based trajectory modeling	BIC Minimum of 5% of participants Posterior probabilities	Linear	STATA proc traj	4	Males 23 40 29 8 Females: 15 35 33 16	No	Measured BMI	Older adulthood (70–79 at baseline)	12	35 years
Salmela, 2020^71^	GBTM	Distinct interpretability Existing literature BIC Posterior probabilities (>0.7) Size of trajectory group (>5%)		STATA traj plug in	4	Stable healthy weight (34) Stable overweight (42) Overweight to class I obesity (20) Stale class II obesity (5)	No	Self‐reported BMI	Midadulthood (40s)–adulthood (60s)	4	17 years
Sayon‐Orea, 2019A^42^	GBTM	BIC Significance of polynomial terms	Cubic and quadratic	STATA traj plug in	4	Males: Lean‐marked increase (29.8) Medium‐marked increase (25.0) Medium‐stable (29.1) Heavy‐stable (16.2) Females Lean‐moderate increase (19.8) Medium‐stable (52.8) Heavy‐medium (21.1) Heavy‐marked increase (6.4)	No	Body shape/somatotype	Childhood (5 years)–midadulthood (40 years)	5	35 years
Sayon‐Orea, 2019B^43^	GBTM	BIC Significance of polynomial terms Average posterior probability (>0.70) Odds of correct classification (>0.5)	Quadratic and cubic	Stata traj plug in	4	Males: Childhood medium‐midlife stable (29) Childhood lean midlife increase (29) Childhood medium‐midlife increase (28) 4Childhood heavy‐midlife stable (14) Females: Childhood medium‐midlife stable (53) Childhood lean‐midlife increase (20) Childhood heavy‐midlife decrease (20) Childhood heavy‐midlife increase (7)	No	Body shape/somatotype	Childhood (5 years)–midadulthood (40 years)	Max of 5 (at age 5, 20, 30, 40, and present) but at least 3 somatotype data	35 years
Song, 2016A^5^	Group‐based modeling	BIC	Cubic	SAS—proc traj	5	Males: Trajectory 1: Lean‐stable (16) Trajectory 2: Lean‐moderate increase (18) Trajectory 3: Lean‐marked increase (38) Trajectory 4: Medium‐stable (15) Trajectory 5: Heavy‐stable/increase (13) Females: Trajectory 1: Lean‐stable (16) Trajectory 2: Lean‐moderate increase (22) Trajectory 3: Lean‐marked increase (21) Trajectory 4: Medium‐stable (27) Trajectory 5: Heavy‐stable/increase (14)	No	Somatotypes	Childhood (ages 5, 10)–adulthood (ages 20, 30, 40, 50, 60)	7	14
Song, 2016B^16^	Group‐based modeling	BIC	Cubic	SAS—proc traj	5	Males: Trajectory 1: Lean‐stable (25) Trajectory 2: Lean‐moderate increase (17) Trajectory 3: Lean‐marked increase (17) Trajectory 4: Medium‐stable/increase (28) Trajectory 5: Heavy‐stable/increase (13) Females: Trajectory 1: Lean‐stable (35) Trajectory 2: Lean‐moderate increase (29) Trajectory 3: Lean‐marked increase (11) Trajectory 4: Medium‐stable/increase (19) Trajectory 5: Heavy‐stable/increase (6)	No	Somatotypes	Childhood (ages 5, 10)–adulthood (ages 20, 30, 40, 50)	6	45 years
Song, 2018^17^	Group‐based modeling (did not specify)	BIC	Cubic	SAS—proc traj	4	Males Lean‐medium trajectory (35) Medium‐medium trajectory (28) Lean‐heavy trajectory (27) Medium‐heavy trajectory (10) Females: Lean‐medium trajectory (35) Medium‐medium trajectory (27) Lean‐heavy trajectory (24) Medium‐heavy trajectory (14)	No	Somatotypes	Childhood (ages 5, 10)–Adulthood (ages 20, 30, 40, 50, 55, 60, 65)	9	16 years
Straughen, 2018^29^	GMM	BIC, AIC Posterior probabilities Group membership	Linear	R—lcmm	4	Group 1: Low‐low (34) Group 2: High‐low (16) Group 3: Low‐high (16) Group 4: High‐high (33)	Yes	Self‐reported BMI (percentiles)	Young adulthood–adulthood (from 18 to 45 years)	3	27 years
Tu, 2015^22^	GBTM	BIC Entropy ranging from 0‐1 Posterior probability (>0.7), High odds of correct classification Biological plausibility	Quadratic, cubic	Stata program developed by jones and nagin	4	Males: Trajectory 1: Low (65.7) Trajectory 2: Decreasing (15) Trajectory 3: Medium (13.4) Trajectory 4: High (5.8) Females: Trajectory 1: Low (46.5) Trajectory 2: Decreasing (8.0) Trajectory 3: Medium (35.4) Trajectory 4: High (10.2)	No	Self‐reported BMI	Childhood–adolescent (1–20 years)	8	16 years (19–35)
VanWagner, 2018^40^	Latent mixture modeling	BIC Group membership (>5%)	N/A	SAS—proc traj	4	Trajectory 1: Stable BMI (26.2) Trajectory 2: Moderate increase (46.0) Trajectory 3: High increase (20.9) Trajectory 4: Extreme increase (6.9)	No	Percentage change in measured BMI relative to baseline	Young adulthood–adulthood (18–60)	8	∼19 years
Viner, 2019^44^	LGMM	AIC Sample‐adjusted BIC Entropy Vuong‐lo‐Mendell‐Rubin test	N/A	Mplus—mixture command	3	Class 1: Normative weight gain (91.6) Class 2: Childhood onset persistent obesity (4.0) Class 3: Adolescent and young adult onset obesity (4.3)	No	Measured BMI at 10 and 16, self‐reported at other ages	Childhood (10, 16) –adulthood (26, 30, 34, 42)	6	32 years
Vistisen, 2014^54^	Latent class trajectory analysis	BIC	Cubic	R—lcmm, hlme function	3	Trajectory 1: Stable overweight (93.6) Trajectory 2: Progressive weight gainers (2) Trajectory 3: Persistently obese (4)	No	Measured BMI	Adulthood (35–55 years of age)	4	Mean 14.1 years (Interquartile range (IQR) 8.7–16.2 years)
Votruba, 2014^37^	LCGM	BIC Posterior probability Group membership (at least 2% to be considered meaningful)	Linear and quadratic	SAS—proc traj	8	Group 1: 10.7 Group 2: 23.1 Group 3: 25.8 Group 4: 16.8 Group 5: 7.8 Group 6: 8.0 Group 7: 5.3 Group 8: 2.7	No	Body weight (kg)	Young adulthood–adulthood (baseline at 18‐24, measured until 45)	At least 4 (range 4–14)	Median 16 years (IQR 11–25 years)
Wang, 2015^67^	LCGM	BIC Value of group membership probability Average posterior probability Significance of polynomial terms	Quadratic, cubic	SAS—proc traj	4	Male: Normal‐stable (31.7) Normal‐overweight (43.6) Overweight‐obese (20.3) Obese‐up (4.4) Female: Normal‐stable (33.7) Normal‐overweight (40.9) Overweight‐obese (18.2) Obese‐Up (7.2)	No	Self‐reported BMI	Young adulthood (20 years) –Midadulthood (55 years)	Up to 9	36 years
Wang, 2016^55^	LCGM	BIC Value of group membership probabliity Average posterior probability Significance of polynomial terms	Quadratic, linear	SAS	4	Trajectory 1: Normal‐stable (23.7) Trajectory 2: Overweight‐stable (45.5) Trajectory 3: Obese‐I‐Stable (24.9) Trajectory 4: Obese‐II‐stable (6.0)	No	Self‐reported BMI	Adulthood–older adulthood (40–70 years)	3 or more On average 5 BMI measures	∼40 years 16–43 years of age
Wang, 2017^62^	LCGM	BIC, Group membership (no less than 5%) Average posterior group membership probabilities (no less than 70%)	Quadratic	SAS	4	Males Trajectory 1: Normal weight‐down (14) Trajectory 2: Overweight‐normal weight (47.9) Trajectory 3: Overweight‐stable (30.5) Trajectory 4: Obese‐stable (7.6) Females Trajectory 1: Normal weight‐down (31.6) Trajectory 2: Overweight‐normal weight (41.9) Trajectory 3: Obese‐I‐Stable (22.3) Trajectory 4: Obese‐II‐stable (4.2)	No	Self‐reported BMI	Older adulthood (65–94 years)	8	14 years
Wijnstok, 2013^36^	LCGA	BIC	Linear	Mplus	S4SF: 2 SFR: 2	Male 2, female 2 for both S4SF and SFR S4SF: Trajectory 1—Favorable: Male 75.9, Female 72.7 Trajectory 2—Unfavorable: Male 24.1, Female 27.3 SFR: Trajectory 1—Favorable: Male 65.5, Female 59.4 Trajectory 2—Unfavorable: Male 34.5, Female 40.6	No	Measured BMI, S4SFs, SFR	Childhood (13 years)–adulthood (42 years)	9	55 years (5–60 years)
Williams, 2017^45^	GBTM	BIC	Quantic and quartic	SAS—proc traj	4	Trajectory 1: Normal (41.6) Trajectory 2: Overweight (404) Trajectory 3: Obese (15.4) Trajectory 4: Morbidly obese (2.7)	No	Measured BMI	Childhood (3, 5, 7, 9, 11, 13, 15, 18 years)–Adulthood (21, 26, 32, 38 years)	9	60 years
Xian, 2017^24^	LCGM) analysis	LMR likelihood ratio test	Linear and quadratic	Mplus	3	Trajectory 1: 50, Trajectory 2: 41, Trajectory 3: 9	No	Measured BMI at baseline, 56, and 62; self‐reported BMI at 40	Young adulthood (20)–adulthood (62)	4	42 years
Yang, 2017^65^	LCGBTM	BIC	Cubic	SAS—proc traj	5	Trajectory 1: Stable normal (36) Trajectory 2: Normal to overweight (46) Trajectory 3: Overweight to obese class I (7) Trajectory 4: Normal to obese class II (8) Trajectory 5: Overweight to obese class III (2)	No	Self‐reported BMI	Young adulthood (18)–adulthood (35, 50–71)	4	32–53 years
Zajacova, 2014^61^	Joint GMM‐DTSA	BIC Adjusted BIC, LMR *p*‐value Bootsrapped Likelihood Ratio Test (BLRT) *p*‐value, entropy index Substantive considerations	Linear	Mplus	3	Male: Trajectory 1: Stable overweight (92) Trajectory 2: Obese declining (3.0) Trajectory 3: Obese losing (4.9) Female: Trajectory 1: Stable overweight (88.4) Trajectory 2: Obese declining (6.7) Trajectory 3: Obese losing (4.9)	Yes (trajectory 3)	Self‐reported BMI	Older adulthood (61–87 years)	8	31 years
Zheng, 2013^56^	Semiparametric group‐based trajectory model	BIC	N/A	SAS—proc traj	6	Trajectory 1: Class II/III obese upward (3.4) Trajectory 2: Class I obese upward (11.7) Trajectory 3: Overweight obesity (22.8) Trajectory 4: Overweight stable (29.5) Trajectory 5: Normal weight upward (24.) Trajectory 6: Normal weight downward (8.4)	Yes (trajectory 6)	Self‐reported BMI	Adulthood–older adulthood (51–77)	9	30 years
Zheng, 2018^68^	Group‐based trajectory modeling (GBTM)	BIC Average group posterior probability	Quadratic	SAS—proc traj	5	Trajectory 1: 12.4, Trajectory 2: 30.0, Trajectory 3: 33.4, Trajectory 4: 18.7, Trajectory 5: 5.5	No	Measured BMI	Young adulthood–older adulthood (17–94)	5	Mean 3.84 SD 0.97 years

Abbreviations: AIC, Akaike's Information Criteria; BIC, Bayesian Information Criteria; BMI, body mass index; CI: Confidence intervals; FIML, full information maximum likelihood; GBTM, group‐based trajectory modeling; GGMM, general growth mixture modeling; GMM, general mixture modeling; GMM‐DTSA, growth mixture‐discrete‐time survival analysis model; LCGA, latent class growth analysis; LCGBTM, latent class group‐based trajectory models; LCGM, latent class growth model; LCGMM, latent class growth mixture modeling; LRT, Likelihood‐ratio test; LMR, Lo‐Mendell‐Rubin; OCC, odds of correct classification; S4SF, sum of four skinfolds; SFR, skinfold thickness ratio; SPMM, semiparametric mixture models.

The number of growth measures used to estimate trajectories ranged from 3 to 16, with a mean of 6.2 (SD = 2.7). The mean trajectory duration (time between first and last anthropometric measurement) was 29.2 years (SD = 17.1). 22 studies that began growth assessment in childhood^5^, ^14^, ^18^, ^20^, ^22^, ^30^, ^31^, ^32^, ^36^, ^38^, ^41^, ^42^, ^43^, ^44^, ^45^, ^46^, ^47^, ^48^, ^49^, ^50^, ^51^ and extended into adulthood. Of these 22 studies with growth trajectories beginning in childhood, five studies^20^, ^22^, ^38^, ^41^, ^51^ had measures until young adulthood (19–23 years old), 12 studies^16^, ^30^, ^31^, ^32^, ^42^, ^43^, ^44^, ^45^, ^46^, ^47^, ^48^, ^49^, ^50^ had measures until midadulthood (38–50 years old), and five studies^5^, ^14^, ^17^, ^18^, ^46^ had measures until older adulthood (60–80 years old). The remaining studies (*n* = 37)^15^, ^19^, ^21^, ^22^, ^23^, ^24^, ^25^, ^26^, ^27^, ^28^, ^29^, ^33^, ^34^, ^35^, ^39^, ^40^, ^52^, ^53^, ^54^, ^55^, ^56^, ^57^, ^58^, ^59^, ^60^, ^61^, ^62^, ^63^, ^64^, ^65^, ^66^, ^67^, ^68^, ^69^, ^70^, ^71^, ^72^ did not include any childhood measures, and only included measures >18 years of age. Of these 37 studies that only reported growth measures in adulthood, six studies^26^, ^61^, ^62^, ^63^, ^64^, ^70^ only included measures during the older adulthood period of life (≥60 years of age).

### 3.3 Statistical approach

As described in Table 3, most studies (*n* = 33) used the statistical software SAS with the procedure *PROC TRAJ* ^73^ to estimate trajectories. Mplus was used by 11 studies and three of these stated *full information maximum likelihood (FIML*) was used, two stated *general mixture modeling* was used, and the others did not specify the specific procedure in Mplus. Only four studies used the statistical software R^74^ to estimate trajectories, and all fit latent class mixed models using the *extended mixed models using latent classes and latent processes* (*lcmm)* package.^75^ The final software that was used to estimate trajectories was STATA, *TRAJ* procedure^76^ (*n* = 11).

### 3.4 Model fit criteria

The studies used various model fit criteria to determine the number of trajectories that optimally fit the data. The most commonly method was the Bayesian Information Criteria, which was used by almost all studies (*n* = 54). Studies also used a combination of the Akaike's Information Criteria, Lo‐Mendell‐Rubin likelihood ratio test, odds of correct classification, posterior probability, significance of polynomial terms, and a priori knowledge to inform the creation of trajectories. Only nine studies included entropy as a criterion for model fit, with the most commonly reported cutoff of >1%. The most common polynomial terms that were found to be significant were quadratic and cubic polynomial terms (Table 3).

### 3.5 Number and naming of identified trajectories

**FIGURE 2:**
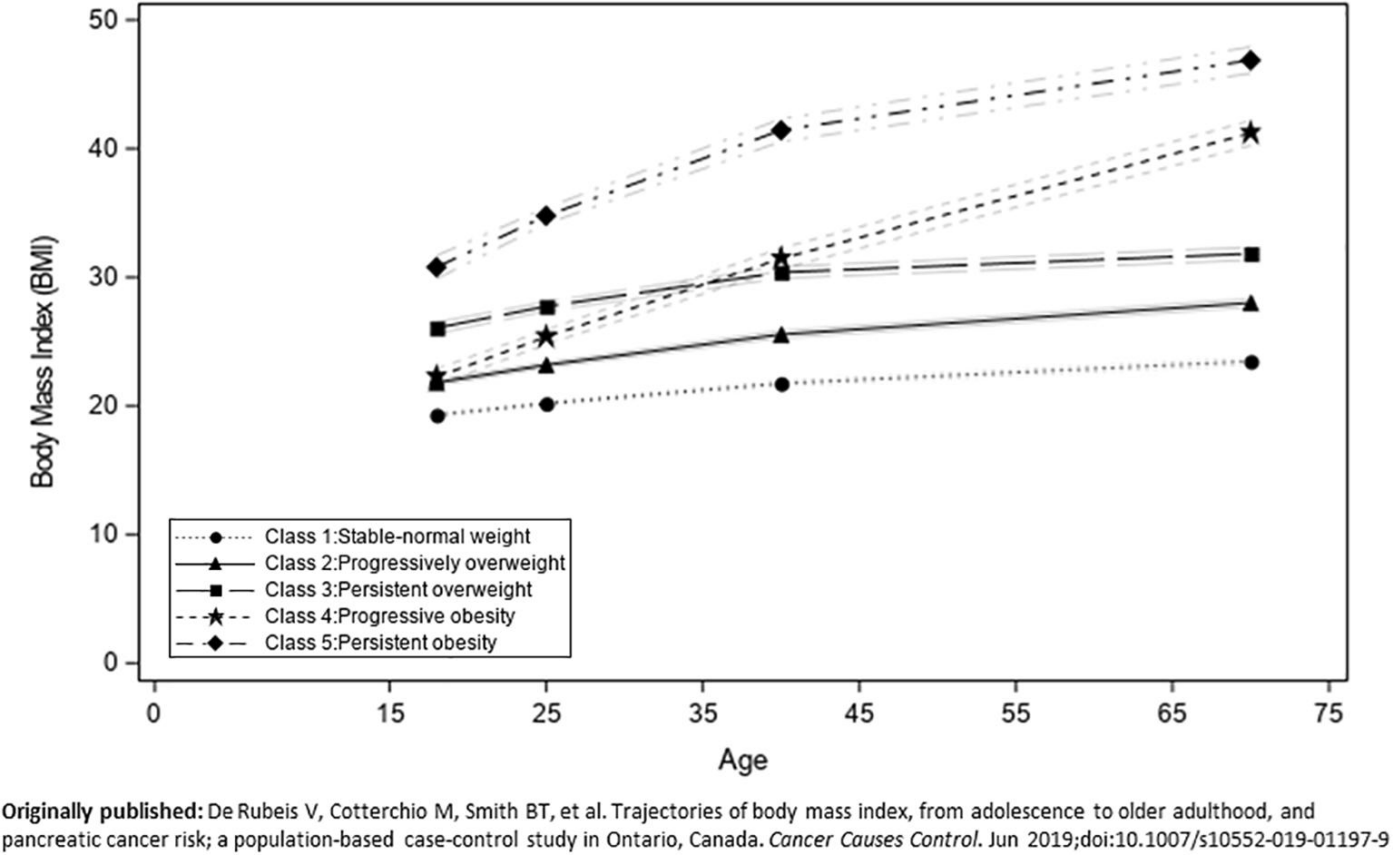
5‐trajectory plot from included study

The number of trajectories that studies identified ranged from 2 to 9. Most studies (56%) found the optimal number of trajectories was 4. Fifteen studies identified five trajectories, and 11 studies identified three trajectories to best fit the data. A sample plot illustrating a 5‐trajectory model from an included study^35^ can be found in Figure 2. The names that were given to the trajectories varied greatly across all studies; however, names were commonly generated based on visual assessments. Most studies used terms such as “normal,” “normative,” “low,” or “stable” to describe the trajectory defined by the lowest weight/BMI throughout the life course. Other common terms used to name trajectories include, “increasing,” “decreasing,” “overweight,” “obese,” and “persistent”. Five studies did not name their identified trajectories, and only referred to the trajectories by group or class number. A detailed description of the various names used to describe the trajectories in each study can be found in Table 3.

The prevalence of the identified trajectories varied, for example the “normal” or “lean stable” trajectory ranged from 14% to 91% of the study populations. Whereas the trajectory with the lowest prevalence was most often the highest growth trajectory, often defined as “persistent obesity” and ranged from 0.8% to 10.5%. 26 studies identified a trajectory defined by a sharp increase in weight throughout the life course and the prevalence of this trajectory varied greatly, ranging from 3% to 29.2%. Only 10 studies identified a trajectory which had a resolving pattern, meaning it began with a higher BMI or body size in earlier life and decreased over the life course and the prevalence ranged from 1.6% to 16%. No studies identified a trajectory characterized by a persistent underweight status.

### 3.6 Sex differences

Of the 59 included studies, 19 studies stratified trajectories by sex and 13 studies only included sex‐specific populations (i.e., males only or females only). Two studies^41^, ^66^ reported they stratified trajectories by sex, however results were not reported since the results were similar to crude analyses. For most studies, the names and number of trajectories were consistent in both men and women, however visual inspection showed slight differences. When comparing trajectories characterized by persistent obesity, the prevalence for males ranged from 0.8% to 16.2%, compared to the prevalence in females, 2.2% to 14%.

### 3.7 Outcome association

A description of the outcomes that were evaluated in relation to BMI/body size trajectories can be found in Table 4. Twenty‐two studies evaluated disease‐related outcomes including cancer, cardiovascular disease, or diabetes. About 15% of studies (*n* = 9) evaluated characteristics associated with trajectories or the association between trajectories and weight later in life. Two studies evaluated maternal trajectories and the association with outcomes in offspring. The remaining trajectories evaluated associations with various outcomes. Although it is difficult to draw conclusions regarding the association between BMI/body size trajectories and disease‐related outcomes due to the heterogeneity of trajectory methodology and outcomes assessed, it appears that trajectories with persistent obesity or trajectories defined by the highest weight throughout the life course typically had the greatest risk of disease related outcomes.

**TABLE 4:** Details of outcomes evaluated in relation to BMI/body size trajectories (*n* = 51)^a^

Outcome evaluated	Number of studies	References
Characteristics BMI trajectories/weight gain	9	19, 20, 22, 37, 52, 53, 63, 64, 67
Cancer	8	5, 25, 27, 30, 31, 35, 59, 65
Cardiovascular disease	8	24, 36, 43, 45, 46, 49, 51, 66
Diabetes	6	32, 33, 54, 68, 72
Mortality	4	16, 56, 61, 62
Overall health outcomes	3	21, 55, 69
Insomnia/sleepiness	2	47, 48
Childhood outcomes	2	28, 29
Genetics and biomarkers	2	14, 18
Other	7	34, 40, 42, 50, 60, 70, 71

Abbreviation: BMI, body mass index.

^a^ Eight studies were excluded since they modeled BMI/body size trajectories as the outcome.

## 4 DISCUSSION

The results from this scoping review suggest that four distinct trajectories of body size across the life course, including adulthood, are commonly identified. This review is the first to evaluate growth trajectories across the entire life course using different anthropometric measures. The findings from this review are consistent with a previous systematic review^41^, ^66^ of trajectories ranging from birth to age 18 only, which found three or four distinct BMI trajectories were most often identified. The average number of growth measures used to identify trajectories was 6.2, with on average 29 years between the first and last anthropometric measures. Most studies did not include a growth assessment during childhood, including only growth measures ≥18 years of age. The methodologies used to identify and estimate trajectories varied across studies in terms of model building approaches, statistical software used, and characteristics of trajectories. Future studies may consider clearly reporting the methodologies used to identify and estimate the trajectories, to allow for transparent reporting.

Due to the heterogeneity of outcomes that were evaluated in relation to the identified trajectories, it was not possible to conduct a meta‐analysis. Although findings varied across studies, it was evident that trajectories defined by the highest body weight or BMI at all ages were most often associated with the greatest risk of disease‐related outcomes. Many studies identified a persistent overweight trajectory and it was generally associated with increased risk, but not always as strongly as the trajectory characterized by persistent obesity. Furthermore, some studies identified a progressive obesity trajectory with lower body size or BMI in childhood or younger adulthood that increased to overweight and obesity later in life, and this trajectory was generally associated with an elevated risk. Only one study identified a trajectory that had high body weight but decreased in later years and no studies identified an underweight trajectory; therefore, making it difficult to comment on the impact of obesity or overweight in childhood or young adulthood as a sensitive period. Future studies that evaluate growth across the life course and disease related outcomes can continue to accumulate evidence identifying sensitive or critical periods of development throughout the life course.^77^ Typically, in epidemiologic research, an exposure is assessed immediately preceding the development of disease. The use of longitudinal data can explain variation in development throughout the life course, and how that may in turn impact the development of an outcome.

Most studies used the statistical approach *PROC TRAJ* in SAS, with only four studies using *lcmm* in R. The low number of studies that utilized the R package may have been because of the recent development of this statistical package.^75^ It is the newest program, which may explain why the least amount of studies used this software. Although the statistical approaches and software are identifying group‐based trajectories, there is limited literature directly comparing each approach. Each program must be downloaded as an additional package or plugin as they are not found in the base program, except the *FIML* procedure in Mplus. All programs use some form of maximum likelihood estimation, which is a general approach for estimating parameters of a probability density function. *TRAJ* in STATA was adapted from *PROC TRAJ* in SAS, and therefore use similar methodologies.^76^ *PROC TRAJ* in SAS uses covariance structure methods, whereas the *lcmm* package in R estimates models with a mixture of linear mixed effects models, allowing for latent classes and random effects to account for repeated measures on subjects.^75^

Although the use of group‐based trajectory modeling is a novel approach, there are several inconsistencies in model building approaches throughout the literature. This limits the generalizability of findings and thus limiting the ability to compare findings across studies. A recent framework by Lennon et al.,^78^ outlines an 8‐step framework which can be used by researchers to ensure group‐based trajectory models are developed in a systematic way. Future studies may benefit from following this framework as it may provide guidance during identification of the most optimal trajectory model. In addition, Guidelines for Reporting on Latent Trajectory Studies (GRoLTS‐Checklist),^79^ which is a 16‐item checklist designed to increase transparently and uniformity of presenting results in latent trajectory studies, was also recently developed. Given that these tools were only recently published, none of the identified studies reported using either the framework or checklist to guide their studies. A formal application of the GRoLTS‐Checklist was beyond the scope of the current project and therefore was not applied. Generally, items addressing the software used, shape and functional forms, metric of time used, and the characteristics of the final class solution were addressed or reported by the studies. Whereas, items surrounding reporting of missing data, information on the distribution of the observed variables and the entropy were not frequently reported. The gaps in reporting following the GRoLTS‐Checklist can inform where future studies When comparing the studies that used the same study populations, it was evident that differences did exist. However, this may be related to differences in the objectives of the studies leading to slightly different participants in the study. For instance, the NLSY was used by five studies. Three of these studies^20^, ^21^, ^22^ reported four trajectories, whereas the remaining two studies reported three^20^, ^21^, ^22^ and five^20^, ^21^, ^22^ trajectories. When the use of the GRoLTS‐Checklist, it would be immediately apparent as to why the different number of trajectories were identified among the same sample and would allow for an easy comparison to be made.

Strengths of this study included the large number of studies that were identified using a systematic search to identify eligible studies. The search strategy was developed, and health research librarians reviewed it to ensure all relevant and necessary search terms were included, and all potentially eligible studies were identified. The broad inclusion of any measure of growth, in terms of BMI and body size, did not limit the identification of any studies that used group‐based approaches to measure growth across the life course. Most studies relied on self‐reported BMI (44%) as the anthropometric measure of growth, which is common in epidemiologic research when resources are limited, and it is not possible to use objective measurements to assess body weight. It has been noted that the use of self‐report of BMI^81^, ^82^ or self‐reported recall of BMI^83^ is a valid measure of true BMI. When evaluating the average number of identified trajectories among studies that used self‐reported BMI and measured BMI separately, the average number of trajectories remains 4 for both methods.

Using life course trajectories provides a much more comprehensive understanding of the impact of differential growth patterns. Group‐based trajectory modeling is a novel approach to identify various patterns of growth throughout the life course. The findings from this review may inform future epidemiologic research on the commonly used methodologies and approaches used to generate group‐based trajectories of growth across the life course. In addition, the use of growth trajectories can be used to inform future public health interventions or prevention strategies targeting subgroups that are at a high‐risk of negative health‐related outcomes.

## CONFLICT OF INTEREST

All authors declare no conflict of interest.

## Supporting information

Supporting Information 1
