# Supplementary material for: Group‐based trajectory modeling of body mass index and body size over the life course: A scoping review
Source: Obes Sci Pract. 2020 Sep 29;7(1):100–28. doi: 10.1002/osp4.456 (PMC7909593; doi:10.1002/osp4.456)
Supplement: Supplementary file 1 — Supporting Information 1 [file OSP4-7-100-s001.docx]

**Appendix**

**Group-based trajectory modelling of body mass index and body size over the life course: a scoping review**

Vanessa De Rubeis^1^, Alessandra Andreacchi^1^, Isobel Sharpe^1^, Lauren E. Griffith^1^, Charles D.G. Keown-Stoneman^2,3^, Laura N. Anderson^1,4^

^1^Department of Health Research Methods, Evidence, and Impact, McMaster University, Hamilton, ON

^2^Applied Health Research Centre of the Li Ka Shing Knowledge Institute of St. Michael's Hospital, University of Toronto, Toronto, Ontario, Canada

^3^Division of Biostatistics, Dalla Lana School of Public Health, University of Toronto, Toronto, Ontario, Canada

^4^Child Health Evaluative Sciences, The Hospital for Sick Children Research Institute, Toronto, ON

**Address/email address:** Laura N. Anderson, PhD, Department of Health Research Methods, Evidence, and Impact, McMaster University, CRL-221, 1280 Main Street West, Hamilton ON L8S 4K1 email: LN.Anderson@mcmaster.ca

**Table A1**. Web of Science search strategy

| 1 | TS=(“latent growth model*” OR “latent class growth mixture model*” OR “growth mixture model*” OR “latent growth model*” OR “latent class growth analysis” OR “latent class growth analyses” OR “group based trajectory model*” OR “group based trajectory analysis” OR “group based trajectory analyses” OR “group based model*” OR “latent growth mixture model*” OR “group based trajectory*”) |
| --- | --- |
| 2 | TS= (growth OR obesity OR “body mass index” OR weight OR “anthropometric parameters” OR “anthropometry”) |
| 3 | 1 and 2 |
|  | 4,698 |

**Table A2**. CINAHL search strategy

| 1 | “latent growth model*” OR “latent class growth mixture model*” OR “growth mixture model*” OR “latent growth model*” OR “latent class growth analysis” OR “latent class growth analyses” OR “group based trajectory model*” OR “group based trajectory analysis” OR “group based trajectory analyses” OR “group based model*” OR “latent growth mixture model*” OR “group based trajectory*” |
| --- | --- |
| 2 | (MH “Growth+”) OR (MH “Body Height”) OR (MH “Body Weight”) OR (MH “Obesity”) OR (MH “Body Weights and Measures+”) |
| 3 | S1 AND S2 |
|  | Limiters – English Language; Human  154 |

**Table A3**. OVID- Medline search strategy

| 1 | “latent growth model*”.mp OR “latent class growth mixture model*”.mp OR “growth mixture model*”.mp OR “latent growth model*”.mp OR “latent class growth analysis”.mp OR “latent class growth analyses”.mp OR “group based trajectory model*”.mp OR “group based trajectory analysis”.mp OR “group based trajectory analyses”.mp OR “group based model*”.mp OR “latent growth mixture model*”.mp OR “group based trajectory*”.mp |
| --- | --- |
| 2 | Exp body size/ OR obesity OR anthropometry/ OR body constitution/ OR exp “body weights and measures”/ OR body weight/ |
| 3 | 1 AND 2 |
| 4 | Limit 3 to (English language and humans) |
|  | 1,761 |

**Table A4.** Detailed characteristics of included studies (n=52)

| **Author, Year, Name of Study** | **Country** | **Study design** | **Sample Size** | **Number of females (%)** |
| --- | --- | --- | --- | --- |
| Adane, 2018, The Australian Longitudinal Study on Women's Health (ALSWH) | Australia | Longitudinal population-based cohort | 1606 | 1606 (100%) |
| Ahanchi, 2019, Tehran Lipid and Glucose Study (TLGS) | Iran | Prospective cohort study | 1823 | 1043 (57.2%) |
| Amadou, 2014, Cancer de mama (CAMA) study | Mexico | Case-control study | 2074 | 2074 (100%) |
| Botoseneanu, 2013, Health and Retirement Study | USA | Longitudinal study | 10314 | 52.30% |
| Buscot, 2018, Young Finns Stud y | Finland | Longitudinal cohort study | 2631 | 1423 (54.1%) |
| Clarke, 2010, Monitoring the Future (MTF) study | USA | Longitudinal study | 5233 | 51.90% |
| Clarke, 2013, Monitoring the Future (MTF) study | USA | Longitudinal study | 10099 | 52.33% |
| Dai, 2019 | China | Longitudinal study | 4519 | 2081 (46.1%) |
| De Rubeis, 2019, Ontario Pancreatic Cancer Study (OPCS) and Ontario Cancer Risk Factor Study (OCRF) | Canada | Case-control study | 1568 | 58.90% |
| Elrashidi, 2016 | USA | Population based | 23254 | 63.10% |
| Elsenburg, 2017, Tracking Adolescents' Individual Lives Survey (TRAILS) | Netherlands | Longitudinal study | 2218 | 1128 (50.9%) |
| Fagherazzi, 2013, Etude Epidémiologique auprès de femmes de la Mutuelle Générale de l’Education Nationale (E3N) study | France | Cohort study | 81089 | 81089 (100%) |
| Fagherazzi, 2015, Etude Epidémiologique auprès de femmes de la Mutuelle Générale de l’Education Nationale (E3N) study | France | Cohort study | 81110 | 81110 (100%) |
| Fan, 2019 | China | Longitudinal study | 3271 | 1559 (46.8%) |
| Hang, 2018, Nurses' Health Study (NHS) | USA | Prospective cohort study | 9613 | 9613 (100%) |
| Ho, 2019, Taiwan Longitudinal Study on Aging | Taiwan | Prospective Cohort Study | 1609 | 778 (48.4%) |
| Huang, 2013, National Longitudinal Study of Youth | USA | Longitudinal study | 5141 | 50.10% |
| Islam, 2019, China Health Nutrition Survey | China | Longitudinal cohort study | 5276 | 2767 (52.4%) |
| Ito, 2020, Japan Nurses’ Health Study | Japan | Prospective cohort study | 7434 | 7434 (100%) |
| Jayne, 2019 | USA | Longitudinal study | 704332 | 98091 (13.9%) |
| Jeon, 2019, Korean Genome and Epidemiology (KoGES) | Japan | Longitudinal study | 4992 | 2650 (53.1%) |
| Jun, 2012, Nurses Health Study II | USA | Longitudinal cohort sequential design | 90713 | 90713 (100%) |
| Kakoly, 2017, Australian Longitudinal Study on Women's Health (ALSWH) | Australia | Community based longitudinal study | 8009 | 8009 (100%) |
| Kelly, 2017, Prostate, Lung, Colorectal, and Ovarian Screening Trial (PLOS) | USA | Population based randomized trial | 69873 | N/A |
| Kuchibhatla, 2013, Duke Established Populations for Epidemiologic Studies of the Elderly Study | USA | Longitudinal study | 3861 | 2495 (64.6%) |
| Kvoerner, 2018, Nurses' Health Study (NHS) and Health Professionals Follow-Up Study (HPFS) | USA | Prospective cohort study | 13327 | 9386 (70.4%) |
| Kwon, 2015, Iowa Bone Development Study | USA | Prospective cohort | 493 | 251 (50.9%) |
| Laddu, 2017, The Osteoporotic Fractures in Men Study (MrOS) | USA | Prospective cohort | 5964 | N/A |
| Lavalette, 2020, EPICAP | France | Case-control study | 1610 | N/A |
| Lisan, 2018, The Paris Prospective Study III | France | Prospective study | 6280 | 2443 (38.9%) |
| Lisan, 2019, The Paris Prospective Study III (PPS3) | France | Prospective study | 7496 | 2886 (38.5) |
| Malhotra, 2013, National Longitudinal Survey of Youth (NLSY79) | USA | Prospective cohort | 10038 | 5001 (49.8%) |
| Nonnemaker, 2009, National Longitudinal Survey of Youth (NLS97) | USA | Prospective cohort | 8984 | N/A |
| Ostbye, 2011, National Longitudinal Survey of Youth (NLSY79) | USA | Prospective cohort | 9681 | 4860 (50.2%) |
| Oura, 2019, Northern Finland Birth Cohort 1966 Study (NFBC1966) | Finland | Prospective cohort | 780 | 418 (53.6%) |
| Petrick, 2017, NIH, ARRP Diet and Health Study and Prostate Lung Colorectal and Ovarian (PLCO) Cancer Screening Trial | USA | Prospective cohort | 409796 | 174628 (42.6%) |
| Reinders, 2015, The Health Aging and Body Composition (Health ABC) Study | USA | Prospective cohort | 998 | 516 (51.7%) |
| Salmela, 2020, Helsinki Health Study | Finland | Prospective cohort study | 7105 | 5790 (82%) |
| Sayon-Orea, 2019A, Seguimiento Universidad de Navarra (SUN) Study | Spain | Prospective cohort | 8891 | 3888 (43.7%) |
| Sayon-Orea, 2019B, Seguimiento Universidad de Navarra (SUN) Study | Spain | Prospective cohort | 7514 | 4490 (59.8%) |
| Song, 2016A, Nurses' Health Study (NHS) and Health Professionals Follow-up Study (HPFS) | USA | Prospective cohort | 106213 | 84792 (69%) |
| Song, 2016B, Nurses' Health Study (NHS) and Health Professionals Follow-up Study (HPFS) | USA | Prospective cohort | 118640 | 81477 (69%) |
| Song, 2018, Nurses' Health Study (NHS) and Health Professionals Follow-up Study (HPFS) | USA | Prospective cohort | 11922 | 7277 (61%) |
| Straughen, 2018 | USA | Cohort study | 1406 | 100% |
| Tu, 2015, National Longitudinal Survey of Children and Youth (NLSCY) and National Population Health Survey (NPHS) | Canada | Cohort study | 7253 | 3560 (49%) |
| VanWagner, 2018, The Coronary Artery Risk Development in Young Adults (CARDIA) Study | USA | Cohort study | 4423 | 1991 (45%) |
| Viner, 2019, British Cohort Study | Britain | Cohort study | 9187 | 4483 (49%) |
| Vistisen, 2014, The Whitehall II Cohort Study | Britain | Cohort study | 645 | 174 (27%) |
| Votruba, 2014 | USA | Longitudinal cohort study | 1157 | 762 (66%) |
| Wang, 2015, National Population Health Survey (NPHS) | Canada | Cohort study | 4790 | 2390 (49.9%) |
| Wang, 2016, National Population Health Survey (NPHS) | Canada | Cohort study | 3070 | 1489 (48.5%) |
| Wang, 2017, National Population Health Survey (NPHS) | Canada | Cohort study | 1480 | 921 (62.2%) |
| Wijnstok, 2013, The Amsterdam Growth and Health Longitudinal Study | Netherlands | Cohort study | 259 | 143 (55.2%) |
| Williams, 2017, Dunedin Multidisciplinary Health and Development (Dunedin) Study | New Zealand | Longitudinal cohort study | 1037 | N/A |
| Xian, 2017, Vietnam Era Twin Study of Aging (VETSA) | USA | Cohort study | 977 | 0 |
| Yang, 2017, NIH-AARP Health Study | USA | Prospective cohort study | 303620 | 126831 (41.8%) |
| Zajacova, 2014, Health and Retirement Study (HRS) | USA | Cohort study | 9703 | 5094 (52.5%) |
| Zheng, 2013, Health and Retirement Study (HRS) | USA | Cohort study | 9538 | 5056 (53.0%) |
| Zheng, 2018, Kailuan study | China | Cohort study | 39321 | 9495 (24.1%) |
